# Bioethical challenges in the implementation of targeted anti-tumor therapies in low-resource settings: a perspective from Latin America

**DOI:** 10.37349/etat.2026.1002397

**Published:** 2026-08-10

**Authors:** Alcides Chaux

**Affiliations:** Fondazione Policlinico Universitario Agostino Gemelli IRCCS, Italy; Agostino Gemelli University Policlinic, Italy; ^1^Facultad de Ciencias de la Salud, Universidad Central del Paraguay, Ciudad del Este 7000, Paraguay; ^2^ChauxLab Institute, Asunción 1230, Paraguay

**Keywords:** targeted therapy, bioethics, Latin America, health equity, precision medicine, distributive justice

## Abstract

Precision oncology has revolutionized cancer care in high-income countries, but its implementation in Latin American low-resource settings faces profound bioethical dilemmas. This study analyzes these challenges through the lens of social justice and equity. An integrative review was conducted following the Whittemore and Knafl framework. A systematic search was performed across PubMed, Scopus, SciELO, and LILACS (2015–2025). Thematic synthesis was applied to integrate empirical data with normative bioethical theories. Four major analytical themes were identified: 1) The innovation paradox and financial toxicity, where prohibitive pricing (exceeding 100,000 USD/year) violates distributive justice and leads to a biological penalty in survival; 2) Infrastructure deficits and epistemic injustice, highlighted by a 9.4% access rate to next-generation sequencing (NGS) and the risks of applying Eurocentric genomic data to admixed LA populations; 3) Research vulnerability, where clinical trials serve as survival strategies, compromising autonomy and informed consent; and 4) The judicialization dilemma, where individual court orders for high-cost drugs threaten systemic sustainability and equity. To prevent a genomic apartheid, Latin America must transition toward genomic sovereignty and frugal precision oncology. Bioethical frameworks in the region must prioritize protection ethics and social justice to ensure that scientific innovation does not exacerbate existing health inequities.

## Introduction

The emergence of precision oncology has revolutionized the therapeutic landscape of cancer, transitioning from non-specific cytotoxic approaches to molecularly targeted therapies that exploit the specific genetic drivers of malignancy. However, while these advancements have established a new standard of care in high-income countries (HICs), their implementation in Latin American low-resource settings presents a complex constellation of bioethical dilemmas that extend beyond traditional medical ethics frameworks [1]. In this region, the clinical application of targeted agents occurs at a contentious intersection of rapid scientific innovation, profound economic constraints, and fragmented healthcare infrastructure, demanding an ethical analysis grounded in social justice and equity rather than individualistic approaches.

The most fundamental bioethical tension in Latin America concerns the prohibitive cost of targeted therapies relative to the financing capacity of public healthcare systems [2, 3]. This creates a structural paradox: while precision medicine is scientifically validated, its market-driven cost structure systematically excludes the very populations that depend on public insurance, thereby violating the principle of distributive justice [4]. Furthermore, the implementation of these therapies is hindered by secondary barriers related to the lack of specialized infrastructure, such as pathology laboratories, genomic profiling capacity, and cold chain management [5]. This infrastructure deficit prevents treatment access and contributes to epistemic injustice, as Latin American populations remain underrepresented in the molecular epidemiologic research that informs international guidelines [6, 7].

Another critical dimension is the ethical paradox of clinical trial participation. Among vulnerable populations in Latin America who are already within the oncology care system, clinical trials may represent the only available access point to innovative treatments. Enrolling these subjects without guaranteed post-trial access (PTA) raises acute concerns regarding exploitation and instrumentalization [8, 9]. Moreover, a deeper stratum of vulnerability exists among those who lack access even to the scientific knowledge or infrastructure that would make trial enrollment a possibility. This situation is further complicated by the phenomenon of medical judicialization—a rapidly escalating trend in Latin America, where the volume of judicial claims for high-cost oncological drugs has grown exponentially over the past decade in countries such as Brazil and Colombia. These court orders bypass systematic resource allocation processes, potentially creating a “Robin Hood” problem that risks institutional bankruptcy and undermines the principle of non-maleficence at a population level [10, 11].

Despite the growing body of clinical literature on targeted therapies, there is a scarcity of integrated analyses that synthesize these bioethical challenges from a specifically Latin American perspective. Most existing frameworks are derived from HIC contexts and may not adequately address the financial toxicity (FT), cultural adaptation of informed consent, or the systemic inequities prevalent in middle-income countries. Therefore, the objective of this integrative review is to analyze the primary bioethical challenges in the implementation of targeted anti-tumor therapies in Latin American low-resource settings, identifying barriers to equity and proposing models to mitigate health inequities in the region.

## Methods

### Study design

This research was conducted as an integrative review, a methodological approach that allows for the simultaneous inclusion of diverse study designs—including experimental, non-experimental, and theoretical research—to provide a holistic understanding of the phenomenon [12]. The review followed the five stages established by the Whittemore and Knafl framework: 1) problem identification, 2) literature search, 3) data evaluation, 4) data analysis, and 5) presentation of results. This design is particularly appropriate for exploring bioethical challenges where empirical data must be synthesized with normative and philosophical perspectives.

### Search strategy and information sources

A systematic search was performed across four electronic databases to ensure global and regional coverage: PubMed/MEDLINE, Scopus, SciELO (Scientific Electronic Library Online), and LILACS (Latin American and Caribbean Health Sciences Literature). The inclusion of SciELO and LILACS was essential to capture evidence specifically generated within and for the Latin American context. The search was limited to articles published between January 2015 and December 2025.

The search strategy employed a combination of Medical Subject Headings (MeSH), Health Sciences Descriptors (DeCS), and free-text keywords. The primary search string was: (“Targeted Therapy” OR “Precision Oncology” OR “Molecular Targeted Therapy”) AND (“Bioethics” OR “Ethics, Medical” OR “Health Equity” OR “Social Justice”) AND (“Latin America” OR “Developing Countries” OR “Low-Resource Settings”). Boolean operators (AND, OR) were used to optimize retrieval. No language restrictions were applied; however, the analysis focused on literature published in English, Spanish, and Portuguese.

### Eligibility criteria

To maintain high scientific rigor, the following criteria were applied.

#### Inclusion criteria

- Peer-reviewed original research (qualitative, quantitative, or mixed methods), systematic reviews, and theoretical or perspective articles focusing on bioethical dimensions.
- Studies addressing targeted anti-tumor therapies (e.g., monoclonal antibodies, tyrosine kinase inhibitors [TKIs], or immunotherapy).
- Research conducted in or focusing on Latin American healthcare systems or low-resource settings with direct regional relevance.
- Articles identifying specific ethical conflicts such as distributive justice, FT, or informed consent in precision oncology.
- Articles published in English, Spanish, or Portuguese.

#### Exclusion criteria

- Purely clinical or pharmacological studies (e.g., drug efficacy reports or Phase I/II trials) without socio-ethical or economic discussion.
- Studies focused exclusively on conventional chemotherapy or radiotherapy.
- Editorials, conference abstracts, and gray literature without formal peer-review validation.

**Figure 1:**
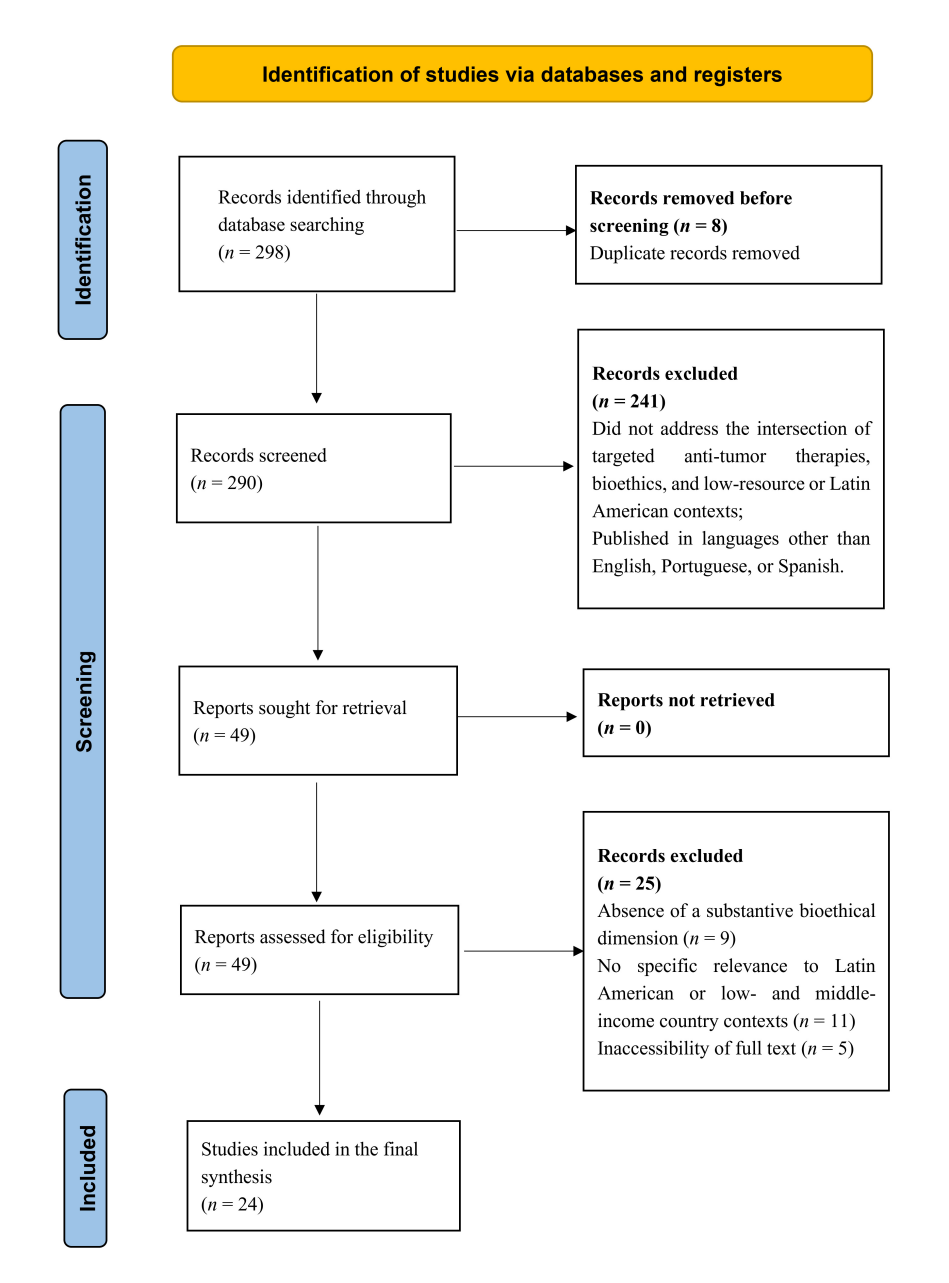
**PRISMA-adapted flow diagram of the search and selection process.** The diagram illustrates the identification, screening, eligibility assessment, and inclusion stages of the integrative review. Adapted from: [13]. © Author(s) 2021. Creative Commons CC BY 4.0 license.

The search and selection process is summarized in Figure 1. A total of 298 records were identified through the database. Following removal of 8 duplicate records, 290 records were screened by title and abstract. Of these, 241 records were excluded as they did not address the intersection of targeted anti-tumor therapies, bioethics, and low-resource or Latin American contexts, or were published in languages other than English, Portuguese, or Spanish. The remaining 49 records were assessed for eligibility through full-text review. Of these, 25 were excluded for the following reasons: absence of a substantive bioethical dimension (*n* = 9), no specific relevance to Latin American or low- and middle-income country (LMIC) contexts (*n* = 11), or inaccessibility of full text (*n* = 5). A final total of 24 studies were included in the integrative synthesis.

A small number of studies conducted in non-Latin American LMICs—specifically India and sub-Saharan Africa—were included where their findings addressed structural barriers, FT mechanisms, or research ethics dilemmas with documented analogues in the Latin American context. The inclusion of these studies was based on the following considerations: 1) the shared structural condition of resource-constrained public healthcare systems with limited molecular diagnostics infrastructure; 2) the comparability of FT mechanisms and drug pricing barriers; and 3) the relevance of their ethical frameworks to the research vulnerability and PTA dilemmas identified in the Latin American literature. It is explicitly acknowledged that these non-Latin American LMIC populations differ from Latin American admixed populations in terms of genetic ancestry composition (Amerindian, African, and European lineages), pharmacogenetic profiles, and healthcare governance structures. Findings from these contexts were therefore incorporated as corroborating structural evidence rather than as direct biological or clinical equivalents, and the transferability of these findings to Latin American populations is acknowledged as a limitation of this review.

### Data evaluation and quality appraisal

The quality of the included studies was appraised using the Mixed Methods Appraisal Tool (MMAT), version 2018 [14], which facilitates the evaluation of diverse methodologies through specific criteria. For theoretical and philosophical papers, appraisal focused on the clarity of the ethical argument and its contextual relevance to Latin America. As this is a single-author review, a two-stage screening process was implemented: the author conducted an initial selection based on titles and abstracts, followed by a second full-text review after a two-week interval to ensure intra-rater reliability and minimize selection bias.

### Data analysis and synthesis

The findings were synthesized using thematic analysis [14, 15]. This process involved: 1) line-by-line coding of the results and discussions of primary studies, 2) grouping codes into descriptive themes, and 3) developing analytical themes that interpret the ethical challenges within the structural constraints of Latin American healthcare systems. This method allowed for the integration of quantitative data on access disparities with qualitative insights into the lived experiences of patients and clinicians.

### Researcher reflexivity

As an integrative review that synthesizes qualitative, quantitative, and theoretical evidence on a value-laden topic, this study is subject to the interpretive influence of the researcher’s positionality. The author is a physician-researcher affiliated with institutions in Paraguay, an LMIC whose healthcare system embodies many of the structural constraints—limited public funding, restricted access to molecular diagnostics, and reliance on judicial mechanisms for access to high-cost drugs—analyzed in this review. This positionality confers a direct contextual understanding of the phenomenon under study and informed the selection of analytically relevant themes. At the same time, it carries the risk of confirmation bias, whereby evidence consistent with the author’s lived professional context may be weighted more heavily than contradictory evidence. To mitigate this risk, the thematic analysis was conducted following explicit coding procedures, the MMAT appraisal was applied using standardized criteria, and interpretive claims in the Discussion are framed with hedging language reflecting the available level of evidence. Readers are encouraged to interpret the findings with awareness of this positionality.

## Results

### Study selection, characteristics and quality assessment

This integrative review included a diverse corpus of literature. The selection comprises original qualitative research, quantitative descriptive studies, mixed-methods designs, and theoretical analyses (Table 1). The evidence primarily originates from major Latin American hubs—including Brazil, Argentina, Mexico, Chile, and Colombia—while also incorporating data from other LMICs with high contextual applicability to the region. The included studies represent a multidisciplinary perspective, incorporating views from oncologists, bioethicists, patient advocacy groups, and health economists, thereby providing a holistic view of the targeted therapy implementation landscape. Theoretical articles were evaluated based on the robustness of their normative arguments, logical coherence, and regional relevance. Overall, the included literature demonstrated high scientific rigor, with approximately 80% of the studies meeting at least four out of five MMAT criteria.

**Table 1:** Characteristics of the studies included in the integrative review (*n* = 24).

**Author(s), year**	**Country/region**	**Study design**	**Main topic**	**Analytical theme**
Penchaszadeh, 2015 [1]	Argentina	Theoretical/perspective	Genetics and public health in Latin America	Research vulnerability
Barrios et al., 2019 [2]	Latin America (Brazil)	Review/perspective	Access to high-cost drugs (trastuzumab) in public systems	Innovation paradox/financial toxicity
Raez et al., 2018 [3]	Latin America (multi)	Review	Access to genomic profiling, immunotherapy, and targeted treatments for lung cancer	Innovation paradox/financial toxicity
de Castilla et al., 2024 [4]	Latin America (multi)	Policy/perspective	Precision oncology implementation: barriers and stakeholder roles	Infrastructure deficits/epistemic injustice
Richard-Greenblatt et al., 2021 [16]	LMIC (global)	Original research	Cold chain logistics for diagnostic sample transport	Infrastructure deficits
Popejoy & Fullerton, 2016 [6]	Global	Data analysis (GWAS Catalog)	Ancestry composition of genomic study participants worldwide	Epistemic injustice
Zavala et al., 2021 [7]	USA/Latin America	Review	Cancer health disparities in racial/ethnic minorities	Epistemic injustice
Mastroleo, 2016 [8]	Global/theoretical	Theoretical/ethical-legal analysis	Post-trial access obligations under the Declaration of Helsinki 2013	Research vulnerability
Andia & Lamprea, 2019 [10]	Colombia/Brazil	Scoping review	Judicialization of healthcare and equity implications	Judicialization dilemma
Riegler, 2023 [11]	Global/theoretical	Theoretical	Comparative ethics of modern payment models and care ethics	Judicialization/financial toxicity
Desai et al., 2022 [17]	USA/LMIC relevance	Original research	Economic cost and sustainability of oral therapies in precision oncology	Innovation paradox/financial toxicity
Elshiekh et al., 2024 [18]	Global	Review	Financial challenges of long-term high-cost medications	Innovation paradox/financial toxicity
Sebastian et al., 2025 [19]	India (LMIC)	Original research	Tepotinib in MET exon 14 skipping NSCLC: real-world LMIC outcomes	Infrastructure deficits/research vulnerability
Kivuyo et al., 2025 [20]	Tanzania (LMIC)	Retrospective review	Pathological response to neoadjuvant chemotherapy in breast cancer	Innovation paradox/infrastructure deficits
Zampoli et al., 2023 [21]	Global/LMIC	Review	Access disparities and ethics of CFTR modulator drugs	Innovation paradox/financial toxicity
Putra et al., 2025 [22]	Asia (LMIC)	Systematic review (abstract)	Socioeconomic inequities in glioblastoma care in Asia	Infrastructure deficits/financial toxicity
Zafar et al., 2013 [23]	USA	Pilot study (quantitative)	Financial toxicity of cancer treatment: out-of-pocket expenses	Innovation paradox/financial toxicity
Smith et al., 2019 [24]	Global	Systematic review	Financial burdens of cancer treatment: risk factors and outcomes	Innovation paradox/financial toxicity
Martínez-Nava et al., 2024 [25]	Latin America (multi)	Systematic review/meta-analysis	Somatic mutations in Latin American breast cancer patients	Epistemic injustice
Zarić et al., 2025 [26]	Global	Review	Pharmacokinetics, pharmacodynamics, and personalized cancer therapy	Epistemic injustice
Corrarino, 2013 [27]	USA/LMIC relevance	Review	Health literacy and women’s health: challenges and opportunities	Research vulnerability
Paasche-Orlow et al., 2003 [28]	USA	Original research	Readability of informed-consent forms vs. actual readability	Research vulnerability
Amerson & Strang, 2015 [29]	LMIC (global)	Review	Research challenges in developing countries: ethical and logistical barriers	Research vulnerability
Salha et al., 2022 [30]	Brazil	Original research (quantitative)	Judicialization of health: oncological medicine demands in Brazil	Judicialization dilemma

Studies classified under Methods [12, 14, 15] served as the methodological framework for this review. LMIC: low- and middle-income country; CFTR: cystic fibrosis transmembrane conductance regulator; NSCLC: non-small cell lung cancer.

Qualitative research successfully addressed the integration of data collection and findings, although a recurring gap was the lack of explicit reflexivity regarding the researcher’s influence, which is particularly sensitive when dealing with vulnerable cancer populations [14]. Quantitative descriptive studies showed strong sampling strategies, though many were limited by non-validated instruments adapted from HICs without prior cultural or linguistic pilot testing. Mixed-methods research exhibited the highest variability in reporting, especially regarding the explicit rationale for integrating qualitative and quantitative strands. Theoretical articles, while not empirically evaluated by MMAT, provided high-level conceptual justifications for applying principles of social justice and distributive ethics to the Latin American landscape, offering essential normative weight to the empirical findings.

The synthesis of the evidence revealed four major analytical themes that define the bioethical landscape of targeted therapies in Latin American low-resource settings [31].

### The innovation paradox: FT and distributive justice

The primary bioethical dilemma identified is the innovation paradox, wherein scientific breakthroughs in precision oncology—designed to improve outcomes—become the very mechanism that exacerbates health inequities in the Global South. This theme explores how the market-driven nature of oncology innovation conflicts with the ethical requirement of universal health coverage.

Targeted therapies, including monoclonal antibodies, TKIs, and immune checkpoint inhibitors, represent a financial impossibility for most Latin American healthcare systems. Illustrative examples include trastuzumab and pertuzumab for HER2-positive breast cancer, osimertinib for EGFR-mutated non-small cell lung cancer (NSCLC), imatinib for BCR-ABL-positive chronic myeloid leukemia, and pembrolizumab for PD-L1-expressing solid tumors—agents whose annual costs routinely exceed the per-capita public health budget of most Latin American countries. Current pricing for many targeted agents exceeds an annual cost of 100,000 to 150,000 USD per patient [17, 18], a burden particularly acute in Latin America where these figures represent 10 to 20 times the annual GDP per capita of the region. These costs far exceed the per-patient oncology expenditure capacity of public healthcare systems [32], creating a structural barrier that effectively excludes the vast majority of the population [3].

Recent data suggest that while these drugs are theoretically available via regulatory approval, their real-world accessibility in public low-resource settings remains below 70%, with access to specific agents for rare molecular alterations (e.g., *MET* exon 14 skipping, *ALK* rearrangements, or *NTRK* fusions) being virtually zero in the public sector [19, 20]. This pricing model ignores the value-based context of the region, where the opportunity cost of funding one patient’s targeted therapy might equal the screening budget for an entire municipality.

The review identifies FT not merely as an economic side effect but as a critical determinant of clinical outcomes and a violation of the principle of non-maleficence. FT forces patients into catastrophic health expenditure, leading to the depletion of family assets, the sale of homes, and the adoption of dangerous coping strategies such as dose-rationing or treatment delay [21].

Quantitative findings highlight that in some LMIC settings, up to 62.5% of patients discontinue targeted therapies prematurely due to cost [22]. This treatment abandonment leads to a biological penalty, where the survival advantage offered by the therapy is lost. For instance, in aggressive malignancies like glioblastoma, median survival can plummet to as low as 7.6 months when supportive and targeted care is inaccessible [22]. Ethically, this situation constitutes structural harm, as the system offers a promise of life that the economic reality subsequently rescinds, resulting in social and economic ruin for the survivor’s family [11, 23].

The evidence underscores a profound public-private dichotomy, manifesting as the “Inverse Care Law”: the availability of high-quality precision oncology is inversely proportional to the medical need of the population [4]. In countries like Brazil and Mexico, the private sector offers molecular profiling and targeted agents comparable to HICs, while public sector patients face therapeutic nihilism—a state where physicians stop discussing innovative options because they know they are unavailable [2]. This disparity creates ethical distress among oncology professionals who navigate the chasm between what is clinically indicated by international guidelines and what is locally possible, often leading to burnout and moral injury [21, 24].

### Infrastructure deficits and epistemic injustice

The implementation of targeted therapies is stymied by secondary barriers related to diagnostic infrastructure. This creates a state of diagnostic abandonment and perpetuates a form of systemic exclusion known as epistemic injustice.

A critical finding is the extreme scarcity of next-generation sequencing (NGS) capabilities in the public sector. Evidence suggested that NGS access in LMIC oncology centers remained critically limited, with available data indicating rates as low as 9.4% [22], a finding consistent with documented barriers to molecular diagnostics infrastructure across low-resource settings, including limited laboratory capacity, absence of bioinformatics expertise, and prohibitive sequencing costs [5]. Without local NGS profiling, actionable alterations such as *EGFR* exon 19 deletions, *ALK* rearrangements, *BRAF V600E* mutations, *NTRK* fusions, and *MET* exon 14 skipping mutations—each with a corresponding approved targeted agent—remain undetected, effectively rendering precision oncology inaccessible regardless of drug availability. This deficit forces reliance on central laboratories abroad or high-cost private labs, introducing logistical delays and a diagnostic odyssey that compromise the window of opportunity for initiating therapy in aggressive malignancies [16, 19]. Furthermore, the lack of cold chain logistics for sample transport often results in degraded biological material, further marginalizing patients in rural areas who are technically eligible but logistically excluded.

The review identifies a profound epistemic injustice rooted in the Eurocentric bias of global genomic databases. Approximately 80% of participants in genomic studies are of European ancestry, which differs significantly from the admixed Latin American populations [6, 7]. This lack of representation means that molecular drivers and somatic mutation profiles in Latin American patients are poorly characterized. This representational injustice can lead to the omission of relevant regional targets or the application of ineffective agents [25]. Ethically, this means that Latin American patients are being treated with proxy data that may not reflect their actual biological reality.

Applying HIC-centric guidelines to Latin American patients carries the risk of unexpected toxicities. Variations in pharmacokinetics and pharmacodynamics (PK/PD) due to ancestral genetic diversity (e.g., variants in CYP450 enzymes specific to Amerindian or African lineages) remain unstudied locally [26]. The principle of non-maleficence is threatened when physicians borrow clinical data from foreign populations, potentially leading to adverse drug reactions or therapeutic failure [25, 26]. There is an urgent ethical imperative to fund local genomic research not as a luxury but as a prerequisite for safe and effective clinical practice.

### Vulnerability in clinical research and informed consent

Structural vulnerabilities in Latin America compromise the pillars of autonomy and justice, turning clinical research into a contested ethical space. Evidence suggests that for many Latin American patients, trial enrollment is a desperation-driven strategy. In contexts where targeted therapies are not funded, trials represent the only access point to innovation [8, 33]. This phenomenon is particularly acute in trials investigating agents such as tepotinib (*MET* exon 14), selpercatinib (*RET* fusions), or entrectinib (*NTRK* fusions), where no publicly funded alternative exists in most Latin American healthcare systems. This therapeutic misconception—where the boundary between research and care is blurred—creates a profound power imbalance. Vulnerable subjects may downplay risks or ignore side effects due to the fear of being removed from a trial that is their only “salvavidas” (lifesaver), raising questions about the true voluntariness of consent and the potential for exploitation [1].

Precision oncology introduces concepts (e.g., germline vs. somatic mutations, probability of response, incidental findings) that exceed the health literacy level of many regional populations. Consent forms often exceed the average patient’s reading ability, being written at a postgraduate level [27, 28]. This literacy gap is acute in indigenous communities where linguistic and cultural differences regarding the concepts of “inheritance” and “illness” prevail. Failure to adapt these forms violates the principle of respect for individuals and turns consent into a bureaucratic signature rather than an autonomous decision [29].

A major ethical conflict identified is the lack of PTA. Responding patients frequently lose access to successful therapies once a trial concludes if the drug lacks local public funding [8, 33]. This constitutes a form of data extraction or biopiracy where the Global South provides the subjects for global approvals, but the participants are denied the long-term benefits of the research. Regional frameworks increasingly advocate for pre-negotiated PTA agreements as a non-negotiable requirement for research approval [33].

### The judicialization dilemma: individual rights vs. systemic sustainability

Medical judicialization—using court orders to compel the state to provide high-cost drugs—challenges the foundations of public health ethics and the principle of equity. The review highlights a tension between the subjective “right to health” (interpreted as the right to any available technology) and the principle of distributive justice. In countries like Brazil and Colombia, judicialization has become a backdoor for high-cost targeted therapies, often bypassing health technology assessments [10]. While court orders fulfill the individual’s claim to life, they frequently ignore the evidence-based prioritization needed to sustain a public system, essentially individualizing a social and political problem [30].

A critical finding is that judicialization frequently exacerbates inequities. Litigants are predominantly individuals with higher educational and socioeconomic levels who possess the legal capital to sue the state [10]. This creates a “Robin Hood” effect in reverse: limited public funds are diverted to cover expensive targeted agents for a few, frequently at the expense of primary oncological services (e.g., HPV vaccination or basic screening) that would benefit the more vulnerable, non-litigating majority [11].

The scale of judicialization threatens systemic non-maleficence. The financial strain can lead to institutional bankruptcy, where the state cuts funding for basic medical supplies to comply with court mandates for a single high-cost drug [30]. This creates a zero-sum game where the benefit to one patient results in harm to the broader population by destabilizing the healthcare infrastructure [11].

Table 2 provides a structured overview of the four analytical themes identified in this integrative synthesis, summarizing key findings, bioethical principles implicated, representative clinical examples, and derived policy implications.

**Table 2:** Structured overview of analytical themes identified in the integrative synthesis.

**Analytical theme**	**Key findings**	**Bioethical principles implicated**	**Representative examples**	**Policy implications**
**1. Innovation paradox and financial toxicity**	Targeted therapy costs exceed 100,000–150,000 USD/year, representing 10–20× the regional GDP per capita; up to 62.5% of patients discontinue treatment prematurely due to cost; financial toxicity leads to catastrophic health expenditure, dose-rationing, and treatment abandonment	Distributive justice; non-maleficence; right to health	Trastuzumab, pertuzumab (HER2+ breast cancer); osimertinib (EGFR+ NSCLC); pembrolizumab (PD-L1+ solid tumors); imatinib (CML)	Tiered pricing models reflecting regional purchasing power; ESMO-MCBS-based prioritization; regional price negotiation treating Latin America as a unified economic bloc
**2. Infrastructure deficits and epistemic injustice**	NGS available in as few as 9.4% of LMIC oncology centers; ~80% of genomic study participants are of European ancestry; admixed Latin American populations (Amerindian, African, European lineages) underrepresented in molecular databases; unstudied CYP450 variants risk unexpected toxicities	Non-maleficence; epistemic justice; scientific integrity	*EGFR*, *ALK*, *BRAF*, *NTRK*, *MET* exon 14, RET alterations undetected without local NGS; Eurocentric genomic databases misrepresent regional mutation profiles	Genomic sovereignty: development of regional reference databases; public investment in local NGS infrastructure; protection of Latin American genomic data from extractive research practices
**3. Research vulnerability and informed consent**	Clinical trials function as survival strategies for patients lacking funded alternatives; therapeutic misconception compromises voluntariness of consent; consent forms written at post-graduate reading level; lack of PTA agreements constitutes biopiracy	Autonomy; justice; respect for persons; non-exploitation	Trials of tepotinib (*MET* exon 14), selpercatinib (*RET* fusions), entrectinib (*NTRK* fusions) with no publicly funded alternative; consent literacy gaps in indigenous communities regarding concepts of inheritance and illness	Mandatory pre-negotiated PTA agreements as condition for research approval; culturally adapted consent through community health mediators and dialogic consent processes; ethics committee reform toward active advocacy
**4. Judicialization dilemma**	Court-ordered access to high-cost drugs bypasses health technology assessment; litigants are predominantly higher socioeconomic status individuals with legal capital; reverse Robin Hood effect diverts public funds from primary oncology services; institutional bankruptcy risk	Justice; equity; non-maleficence at population level; sustainability	Judicialization of trastuzumab, bevacizumab, and checkpoint inhibitors in Brazil and Colombia; diversion of funds from HPV vaccination and basic screening programs	Frugal precision oncology paradigm; strengthening of HTA processes incorporating ethical values; legal frameworks distinguishing individual rights from systemic equity obligations

CML: chronic myeloid leukemia; ESMO-MCBS: European Society for Medical Oncology Magnitude of Clinical Benefit Scale; HTA: health technology assessment; LMIC: low- and middle-income country; NGS: next-generation sequencing; NSCLC: non-small cell lung cancer; PTA: post-trial access.

## Discussion

This integrative review demonstrates that the implementation of targeted anti-tumor therapies in Latin American low-resource settings is not merely a logistical or financial hurdle, but a profound bioethical crisis that challenges the foundations of global health equity. The findings suggest that the current trajectory of precision oncology, if left unmitigated by regional ethical frameworks, may threaten to transform genetic innovation into a new instrument of social stratification, creating a genomic divide that mirrors existing socioeconomic fissures. We propose the term “genomic apartheid” to describe this structural condition: a system in which access to genomic medicine—including molecular diagnostics, targeted therapies, and genomic research participation—is systematically stratified along socioeconomic and geographic lines, resulting in differential survival outcomes determined not by tumor biology but by place of birth and purchasing power. While this term does not yet appear as a formally codified construct in the bioethics literature, the underlying phenomenon is extensively documented. The genomic divide between high-income and low-income countries has been identified as a critical threat to global health equity [34, 35], with genomic research authorship and data generation remaining overwhelmingly concentrated in high-income settings [36], and international organizations explicitly warning that without deliberate intervention, genomic innovation will deepen rather than reduce existing health inequities [37].

The innovation paradox identified in this review reveals that targeted therapies are currently operating within a framework of distributive injustice that can be characterized as a form of structural violence. While international guidelines, predominantly authored in HICs, emphasize the biological superiority and necessity of targeted agents, market-driven pricing models [17, 18] systematically ignore the macroeconomic reality of Latin American healthcare systems. This creates a state of structural harm: the medical system successfully identifies a life-saving molecular target but remains ethically paralyzed by the inability to provide the corresponding therapy.

The consequences of this paradox are devastatingly tangible. The biological penalty of 7.6 months of median survival observed in settings with restricted access is not a result of tumor biology but of political and economic choices [22]. Ethically, this requires a paradigm shift from a purely individualistic, autonomy-based bioethics toward a “protection bioethics” [1]. In this framework, the moral responsibility for health outcomes should be redistributed: the state must act as a guarantor of access, and the pharmaceutical industry must acknowledge its role in the “right to science” by adopting tiered pricing models that reflect local purchasing power rather than global profit maximization.

A central contribution of this review is the identification of epistemic injustice as a pervasive barrier to safe and equitable care. By relying on Eurocentric genomic databases—where approximately 80% of participants are of European descent [6, 7]—Latin American oncology may inadvertently be practicing a form of proxy medicine. Applying HIC-derived evidence to highly admixed populations (comprising Amerindian, African, and European lineages) without local validation constitutes a violation of the principle of non-maleficence. The unstudied pharmacogenetic risks associated with regional genetic variants (e.g., CYP450 polymorphisms) mean that Latin American patients may be subjected to suboptimal dosing or unexpected toxicities [25, 26].

To mitigate this, we propose the concept of “Genomic Sovereignty” as an ethical and strategic imperative. This goes beyond merely filling the NGS gap—which currently leaves 90.6% of centers in low-resource settings in a state of molecular blindness [22]. Genomic sovereignty demands that the region develops its own reference databases and that the genomic data of Latin American citizens are protected from biopiracy or data extraction by HIC-based entities without guaranteed local clinical translation. Precision medicine in the Global South must be locally grounded to be ethically defensible.

The results underscore that clinical trials frequently serve as the only access point for innovative therapies in Latin America [8]. This vulnerability of necessity creates a profound ethical tension. When a clinical trial is a patient’s only lifeline, the voluntariness of informed consent becomes a bureaucratic fiction rather than a robust autonomous choice. Patients in this state of therapeutic desperation are prone to the therapeutic misconception, downplaying significant risks in exchange for the hope of survival.

Furthermore, the lack of robust PTA agreements in the region represents a glaring failure of justice. Ethical committees must transition from passive observers to active advocates for the social value of research. A trial should only be deemed ethical in a low-resource setting if it includes a transparent, pre-negotiated pathway for the long-term sustainability of the intervention for those who benefited from it [9]. Without PTA, clinical research may risk becoming a form of ethics dumping, where the Global South provides the clinical data while the Global North reaps the therapeutic benefits [1, 8, 33].

The phenomenon of medical judicialization [10, 30] must be interpreted as a symptomatic reaction to the failure of health technology assessment processes to incorporate ethical values. While court orders fulfill the individual’s “right to life,” they often pervert the principle of equity. The “Robin Hood effect” in reverse—where limited public funds are diverted to cover high-cost agents for the few who possess the legal capital to sue—further marginalizes the non-litigating majority [11, 30].

Rather than focusing on legal suppression, healthcare systems must move toward a frugal precision oncology. This paradigm involves: 1) prioritizing targeted therapies with the highest clinical benefit-to-cost ratio (e.g., those targeting “A” or “B” scores on the ESMO-MCBS scale); 2) investing in local diagnostic infrastructure to ensure “precision” is actually achieved; and 3) regional price negotiations that treat Latin America as a unified economic bloc. This approach protects institutional sustainability while fulfilling the moral obligation to provide evidence-based care [38, 39].

This integrative review possesses several strengths that distinguish it from existing literature. First, by adopting the framework of Whittemore and Knafl [12], we were able to synthesize diverse evidence types—ranging from hard quantitative statistics on survival to qualitative insights into the lived experience of FT—providing a multidimensional view of the crisis. Second, the explicit focus on Latin American admixed populations highlights a specific form of epistemic injustice often overlooked in global bioethical discourses. Third, the inclusion of regional databases like SciELO and LILACS ensured that the voice of Latin American researchers was central to the analysis, rather than relying solely on indexed literature from HICs. Finally, the use of the MMAT (2018) for quality appraisal provides a high level of methodological transparency and rigor, reinforcing the reliability of our findings for policy-making.

Despite its strengths, this review is limited by the inherent heterogeneity of the included studies and the volatile nature of oncology drug pricing, which may alter the FT landscape rapidly. A primary methodological limitation of this review is its single-author design. Standard integrative and systematic reviews recommend the involvement of at least two independent reviewers for both study selection and quality appraisal, as this ensures inter-rater reliability and reduces the risk of subjective bias in inclusion decisions and MMAT scoring. In the present review, this limitation was mitigated through a two-stage screening process: an initial selection based on titles and abstracts was followed by a second full-text review conducted after a two-week interval, allowing intra-rater consistency to serve as a partial proxy for inter-rater agreement. MMAT appraisal was conducted using the standardized criteria and decision rules specified in Hong et al. [14], minimizing discretionary judgment. Nonetheless, the possibility of undetected selection or appraisal bias cannot be excluded, and findings should be interpreted with this constraint in mind.

A further limitation concerns the selection of databases. Although PubMed, Scopus, SciELO, and LILACS were chosen to optimize both global and regional coverage, other relevant databases—including Web of Science, Embase, and CINAHL—were not searched. It is acknowledged that some pertinent literature indexed exclusively in these sources may not have been captured. However, a substantial proportion of the oncology and bioethics literature indexed in Web of Science and Embase overlaps with PubMed and Scopus, and the manual reference list screening performed as a complementary strategy provided an additional safeguard against significant omission. The reviewed literature is therefore considered to represent a robust and regionally relevant body of evidence for the purposes of this integrative synthesis.

Future research should focus on developing culturally adapted informed consent models that utilize visual aids and plain language to bridge the health literacy gap [27, 28]. Two specific strategies merit prioritization in indigenous community contexts. First, the co-development of consent materials with community health mediators—bilingual individuals embedded within the community who can translate not only language but conceptual frameworks, reframing genomic concepts such as “inherited mutation” in terms of culturally resonant notions of lineage, ancestry, or collective identity rather than individual biological determinism. Second, the adoption of dialogic consent processes, replacing the signature of a written form with a structured oral dialogue—ideally recorded with the participant’s consent—that allows the researcher to verify genuine comprehension of key concepts, including the distinction between somatic and germline alterations and the meaning of probabilistic risk. These approaches are consistent with emerging frameworks for ethical research in indigenous populations and directly address the autonomy deficit identified in this review [29].

## Conclusions

The implementation of targeted anti-tumor therapies in Latin American low-resource settings represents a profound bioethical crisis that extends well beyond logistical or financial constraints. This integrative review identified four interconnected dimensions of this crisis: the innovation paradox and FT generated by market-driven pricing models incompatible with regional purchasing capacity; infrastructure deficits and epistemic injustice perpetuated by the Eurocentric bias of global genomic databases; the structural vulnerability of patients who enroll in clinical trials as a survival strategy, often under conditions that compromise genuine informed consent; and the judicialization dilemma, where individual court-ordered access to high-cost therapies threatens systemic equity and institutional sustainability.

To prevent the consolidation of a genomic apartheid—where survival is determined by a combination of genetic ancestry and socioeconomic status rather than clinical need—Latin America must advance toward genomic sovereignty and frugal precision oncology. Bioethical frameworks in the region must prioritize protection ethics and social justice, ensuring that scientific innovation serves as a tool for reducing, rather than exacerbating, existing health inequities. Innovation without equitable access is not progress; it is a violation of the fundamental right to health.

## Abbreviations

FT: financial toxicity
HICs: high-income countries
LMIC: low- and middle-income country
MMAT: Mixed Methods Appraisal Tool
NGS: next-generation sequencing
PTA: post-trial access
TKIs: tyrosine kinase inhibitors

## Declarations

### Availability of data and materials

The primary data for this systematic review were sourced online from databases listed in the methods. Referenced articles are accessible on PubMed/MEDLINE, Scopus, SciELO (Scientific Electronic Library Online), and LILACS (Latin American and Caribbean Health Sciences Literature). Additional supporting data are available from the corresponding author upon request.

### Copyright

© The Author(s) 2026.
